# A Semi-Automated Object-Based Gully Networks Detection Using Different Machine Learning Models: A Case Study of Bowen Catchment, Queensland, Australia

**DOI:** 10.3390/s19224893

**Published:** 2019-11-09

**Authors:** Hejar Shahabi, Ben Jarihani, Sepideh Tavakkoli Piralilou, David Chittleborough, Mohammadtaghi Avand, Omid Ghorbanzadeh

**Affiliations:** 1 Department of Remote Sensing and GIS, University of Tabriz, Tabriz 5166616471, Iran; h.shahabi94@ms.tabrizu.ac.ir; 2 Mountain Societies Research Institute, University of Central Asia, Khorog 736000, Tajikistan; bjarihan@usc.edu.au (B.J.); david.chittleborough@adelaide.edu.au (D.C.); 3 Sustainability Research Centre, University of the Sunshine Coast, Sunshine Coast, QLD 4556, Australia; 4 Department of Geoinformatics-Z _GIS, University of Salzburg, 5020 Salzburg, Austria; sepideh.tavakkoli-piralilou@stud.sbg.ac.at; 5 School of Physical Sciences, University of Adelaide, Adelaide 5005, Australia; 6 Faculty of Natural Resources and Marine Sciences, Tarbiat Modares Unviversity (TMU), Tehran 46414-356, Iran; mt.avand70@gmail.com

**Keywords:** geographic object-based image analysis (GEOBIA), gully erosion, optimal scale detection, stacking model, Bowen catchment

## Abstract

Gully erosion is a dominant source of sediment and particulates to the Great Barrier Reef (GBR) World Heritage area. We selected the Bowen catchment, a tributary of the Burdekin Basin, as our area of study; the region is associated with a high density of gully networks. We aimed to use a semi-automated object-based gully networks detection process using a combination of multi-source and multi-scale remote sensing and ground-based data. An advanced approach was employed by integrating geographic object-based image analysis (GEOBIA) with current machine learning (ML) models. These included artificial neural networks (ANN), support vector machines (SVM), and random forests (RF), and an ensemble ML model of stacking to deal with the spatial scaling problem in gully networks detection. Spectral indices such as the normalized difference vegetation index (NDVI) and topographic conditioning factors, such as elevation, slope, aspect, topographic wetness index (TWI), slope length (SL), and curvature, were generated from Sentinel 2A images and the ALOS 12-m digital elevation model (DEM), respectively. For image segmentation, the ESP2 tool was used to obtain three optimal scale factors. On using object pureness index (OPI), object matching index (OMI), and object fitness index (OFI), the accuracy of each scale in image segmentation was evaluated. The scale parameter of 45 with OFI of 0.94, which is a combination of OPI and OMI indices, proved to be the optimal scale parameter for image segmentation. Furthermore, segmented objects based on scale 45 were overlaid with 70% and 30% of a prepared gully inventory map to select the ML models’ training and testing objects, respectively. The quantitative accuracy assessment methods of Precision, Recall, and an F1 measure were used to evaluate the model’s performance. Integration of GEOBIA with the stacking model using a scale of 45 resulted in the highest accuracy in detection of gully networks with an F1 measure value of 0.89. Here, we conclude that the adoption of optimal scale object definition in the GEOBIA and application of the ensemble stacking of ML models resulted in higher accuracy in the detection of gully networks.

## 1. Introduction

In most soil erosion studies, erosion by water is considered a natural part of the geomorphological cycle of the earth system which has destructive impacts on farming, land, and infrastructure. In some cases, the effects are irreparable, especially concerning loss of fertile soil and dam reservoirs sedimentations [1,2]. Every year water erosion delivers massive amounts of sediments to dams, estuaries, and near-shore marine environments [3] threatening inland and aquatic ecosystems. Of the four forms of soil erosion—sheet, rill, splash, and gully—gully erosion is recognized as a natural event that affects farmlands, infrastructures, and aquatic ecosystems [4,5,6].

The gully is defined as a waterway that can be developed from rill erosion; it is relatively permanent with vertical walls on its sides that temporary currents of water pass through. In the long term, soil erosion causes these gullies to change the landscape, resulting in land degradation and geomorphic changes to river systems [7,8]. Gullies can cause a variety of environmental and socio-economic issues, such as decrease in soil productivity and damage to infrastructure [9,10], and in some cases, landslides [11]. In Australia, especially in Queensland, gully erosion is one of the main geo-environmental degradation processes resulting from excessive land clearing, inappropriate land use, and high-intensity rainfall [12,13]. Sediment and the nutrients associated with it is one of the key factors threatening the health of the Great Barrier Reef (GBR) [14,15,16]. A study in 2012 reported that the Burdekin catchment is the primary source of sediment to the GBR—it deposits almost 4 M tons of sediment per year [17]. Within the Burdekin catchment, the Bowen sub-catchment has been identified as the primary source of sediment load to GBR that is associated with a high density of gully networks [18,19]; hence, there is the need to detect and predict where gully erosion networks form and expand in the landscape. Our ability to accurately quantify sediment loads contributed by catchments, identify erosion hotspots, and prioritize and target management interventions is currently constrained by a lack of information—at an optimal scale—on both locations, density, and extent of gully systems.

In this regard, many expert knowledge-based and machine learning (ML) models, including analytical hierarchy process (AHP), logistic regression (LR) [20,21], weights of evidence (WoE) [22], frequency ratio (FR) [23], multivariate adaptive regression splines (MARS) [24], and random forest (RF) [25] have been applied to generate gully erosion susceptibility maps. Besides, some ML models, including artificial neural network (ANN), support vector machines (SVM), and RF have been used for both detection and susceptibility mapping of landslides and gully networks in various studies [25,26,27]. The main reasons why ML models are widely used in various scientific fields, especially in geosciences, are their remarkable performance, flexibility, and accuracy in modeling and predicting phenomena [28,29,30], whereas expert knowledge-based models such as GIS-based multi-criteria decision making (MCDM) depend highly on an expert’s judgment and are associated with uncertainty [31,32,33]. In ML models, detection of gully networks is mostly carried out at a pixel level [34,35,36]. Over the last two decades, geographic object-based image analysis (GEOBIA) has been reported as an efficient and robust method in the image analysis domain, especially for detection of landslides and gully networks [37,38,39,40]. The GEOBIA system imitates human perception, thereby attempting to create real-world objects from pixels that share similar values [41,42]. The primary step in the GEOBIA system is image segmentation. Although no segmentation process is perfect, it needs to be done precisely because non-optimal segmentation does not result in the best classification. In pixel-based methods, image analysis highly depends on pixel values, whereas in the object-based method, other descriptions such as spectral information and spatial properties, together with textural data and contextual information, are considered [43]. These descriptions can then be applied to feature annotation or image classification. The critical issue in object-based methods is how a “meaningful” description can be defined for any feature detection and classification at multiple scales [44]. Although some segmentation solutions have been reported by researchers [45], the definition of the optimal spatial scale for object definition regarding each specific spatial problem such as gully networks detection is still unclear. Image segmentation in gully and landslide detection studies has been undertaken by visual interpretation or trial-and-error [46].

In this study, we integrate the GEOBIA segmentation at different scales with ML models and call it a semi-automated object-based approach to detect gully networks. Based on the results of the ML models, the optimal scale of segmentation was selected and used for further analysis. Besides conventional ML models, another ensemble ML model, namely stacking, which has not been used in gully mapping, was also tested. The stacking model combines different ML models in order to build a robust prototype for prediction and classification proposes [47]. Due to lack of gully inventory data for the whole Bowen catchment, a specific region in the northwest of the catchment that has a high rate of gully features was chosen to evaluate our methodology for gully networks detecting.

## 2. Study Area

The Bowen catchment is associated with a high density of gully networks and has been identified as the primary source of sediment load to GBR. According to Köppen Climate Classification, this catchment is located within Central Queensland in a tropical wet-and-dry climate (AW). Average annual precipitation and temperature are 978 mm and 24.2 °C, respectively. The region in this study is located northwest part of the catchment, and its extent in UTM 55 s zone is between (542260 m–558650 m X, 7716420 m–7716420 m Y).

## 3. Methodology

### 3.1. Overall Workflow

The workflow of the present study is as follows:

- Preparing Sentinel 2A images, spectral index, and conditioning factors for modelling
- Preparing training and validation for recognition of gully features by
- Running image segmentation using the ESP2 tool
- Assessing the accuracy of produced objects using different scale values
- Selecting the optimal scale value
- Training and testing the ML models based on a gully inventory map
- Selecting the best ML model based on the resulting maps

### 3.2. Datasets

**Figure 1:**
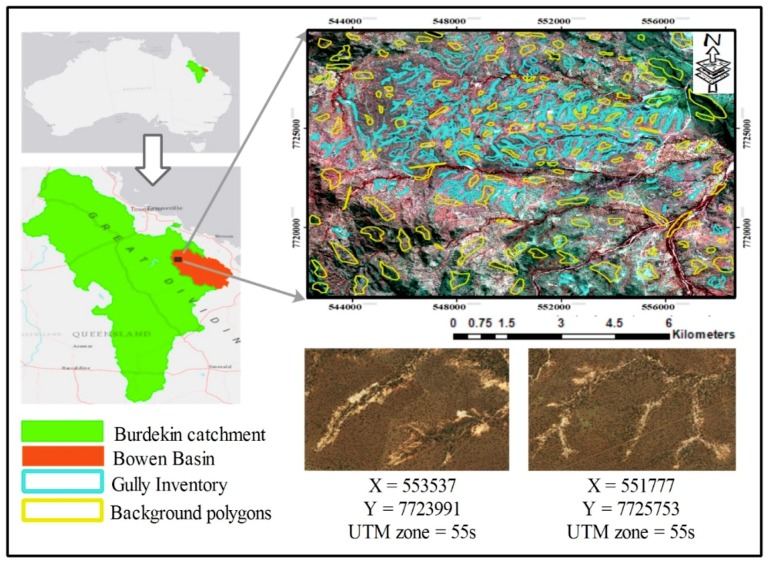
Location of study site in the Bowen Catchment. The background images are Sentinel 2A 10-m resolution false color composite and from Google Earth.

**Figure 2:**
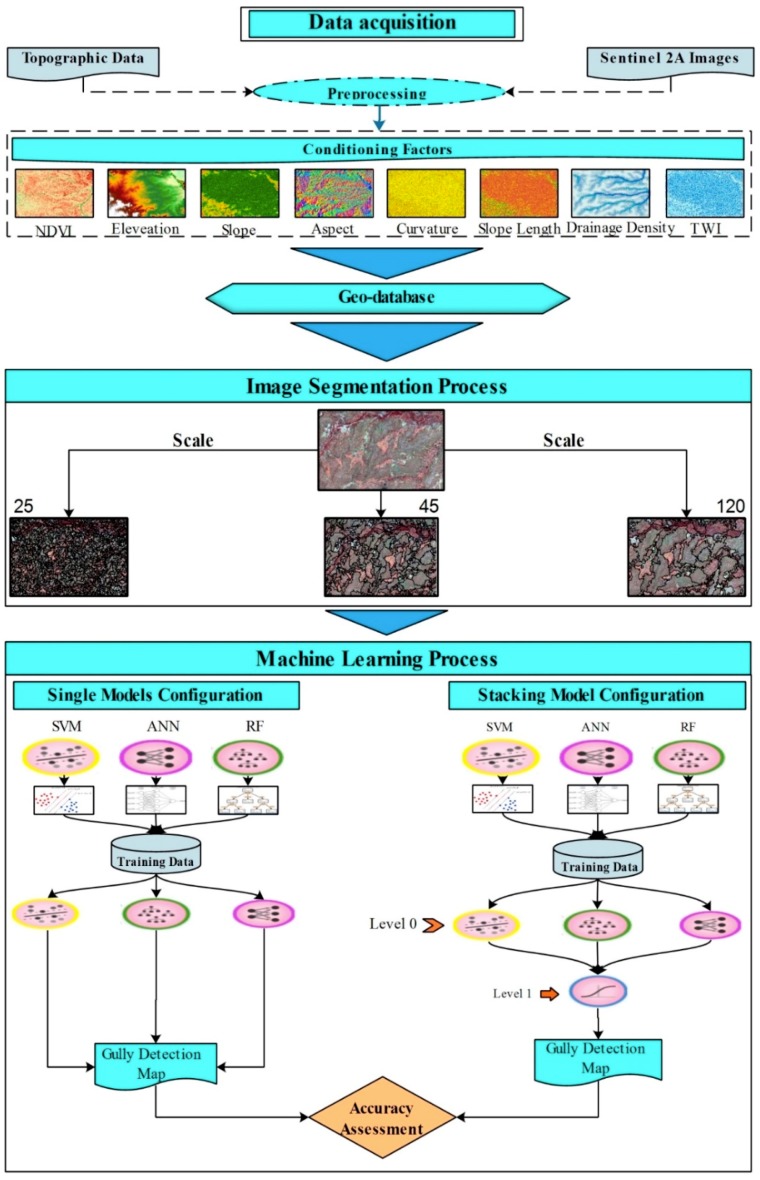
Flowchart of the methodology used in the current study.

**Figure 3:**
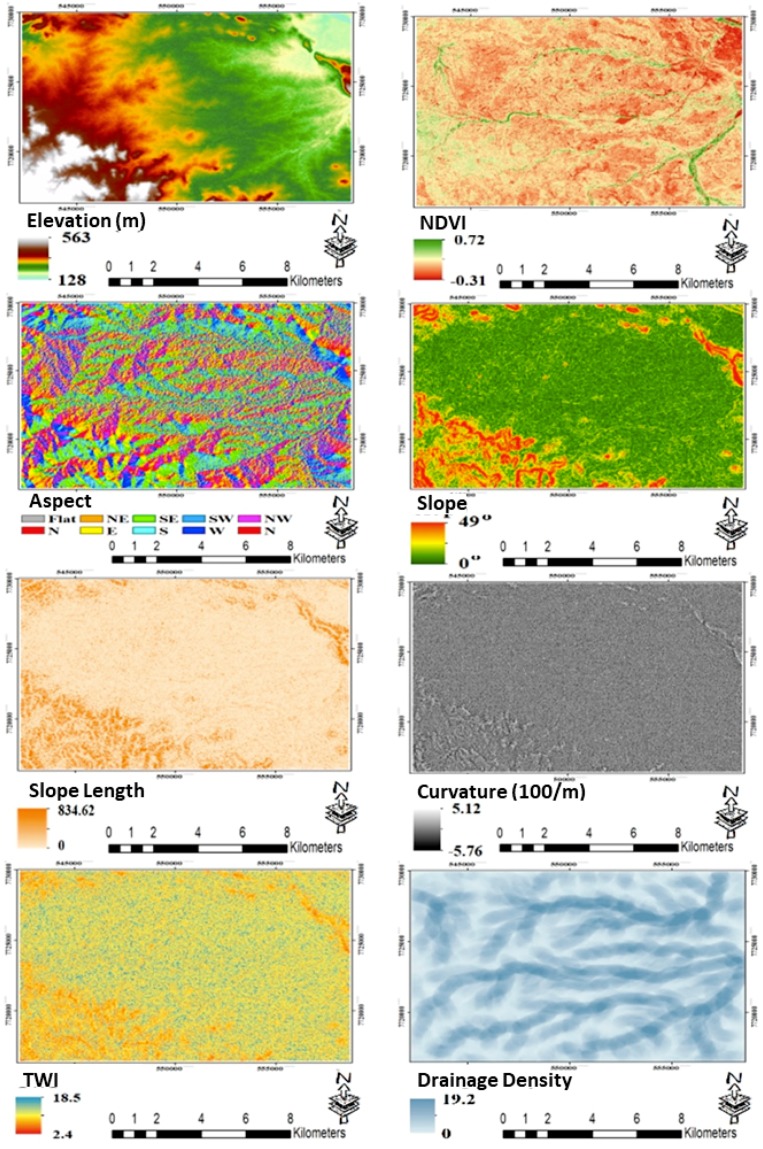
Conditioning factors used for gully networks detection.

ML models and image classification algorithms require a set of valid inventory data for training and validation processes. Thus, the gully inventory map (112 polygons) and background data (136 polygons) in our study (Figure 1) were digitized by the GIS and remote sensing group at the Department of Engineering and Science, University of the Sunshine Coast, using high-resolution images from Google Earth and PlanetScope satellites. Almost 70% of the gully inventory map was labelled as training data, and the other 30% was used for validation of results (Figure 2). For background class data, we randomly selected 200 non-gully objects of various land covers that were overlapped with background polygons. Furthermore, Sentinel 2A data (Level-1C) with 10-m spatial resolution were downloaded for 3 September 2018. The Sentinel 2A Level-1C product includes images for top of atmosphere (TOA) reflectance values, and using Sen2Cor plugin in SNAP 6.0 software, TOA values were converted into bottom of atmosphere (BOA) reflectance values, which is called Level-2A product. The preprocessed Sentinel 2A images were masked and used to calculate the normalized difference vegetation index (NDVI) and implement image segmentation in eCognition software. In forming and controlling gullies, topographic characteristics—in particular, drainage density and slope—play an important role by affecting run-off rate and amount of surface water [48,49,50,51], and they have been widely used in gully erosion susceptible mapping [26,48,52,53] and gully network detection [38,50,54]. Therefore, in this study, in addition to satellite images and NDVI vegetation index, a series of topographic factors (Figure 3), such as elevation, slope, slope aspect, slope length, drainage density, and topographic wetness index (TWI) were used. These parameters were extracted from ALOS-2 DEM with a 12-m spatial resolution, which is produced from ALOS-2 images captured on 22 August 2018. DEM data was obtained from the Queensland Spatial Catalogue (www.qldspatial.information.qld.gov.au) on 27 September 2018. Since the time difference between acquisition of ALOS-2 data and Sentinel 2A is very short, we do not expect any considerable changes that might affect the results. Moreover, there have not been any significant rainfall events in that period to cause considerable geomorphic changes, as flood and heavy rainfalls can substantially change or generate new gullies [3,55].

### 3.3. Geographic Object-Based Image Analysis (GEOBIA)

GEOBIA concepts and approaches have been applied widely in several domains of image classification and object annotation [46]. Image segmentation is considered the most critical process in GEOBIA to address problems that are associated with conventional pixel-based image processing methods. In the pixel-based approaches, image processing relies on the value of pixels, a reliance that limits its applications. On the other hand, object-based image analysis is not limited to pixel value, but applies characteristics including size, shape, texture, color, and contextual information in order to classify or detect desired features [41,42,43,44,45,46,47,56]. In an extensive literature review, GEOBIA is considered a knowledge-driven method that is designed to create a set of homogeneous image objects that reveal current real-world features [37,38,39,40,41]. Given that the most critical stage in the object-based method is image segmentation [56], selection of segmentation parameters and scales affect, to a significant degree, the size and shape of image objects, and incorrect parameters result in under-segmentation and over-segmentation errors.

In this study, we applied a multi-resolution segmentation (MRS) method in eCognition 9.1. However, the ESP2 tool proposed by Drăguţ et al. [45] was used to select the optimal scale. This tool, which was developed for the eCognition Developer software, presents three separate scale (fine to coarse) values using the MRS method based on a set of given parameters. To select the optimal scale using the ESP2 tool, three preliminary scales need to be introduced; in this case, we used 10 for level 1, 20 for level 2, and 30 for level 3 with shape and compactness value of 0.5. The ESP2 tool resulted in three fine, moderate, and coarse-scale values of 25, 45, and 120, respectively. Furthermore, to evaluate the accuracy of each proposed scale value in image segmentation, three geometrical and spectral indices—Object Pureness Index (OPI), Object Matching Index (OMI), and Object Fitness Index (OFI)—were used. The indexes are fully described by Tavakkoli et al., 2019 [57]. In OPI, object integrity in terms of spectral characteristics is assessed. Due to a strong positive correlation between the Sentinel-2 bands (blue, green, red), the standard deviation (SD) values of an object in blue, green, and red bands are quite close, while mean values are not. For perfect and homogenous objects (e.g., water bodies), SD values of the mentioned bands are similar; however, for heterogenous objects, which usually are produced by higher scale values, SD values substantially vary. Another applied index, OMI, assesses the quality of produced objects in terms of spatial matching in accordance with reference objects. The mathematical expression of OPI and OMI is as follows [57]:

(1) $$\mathrm{OPI}\;=\;\frac{\frac{{(\mathrm{SD}}_{B}{+\;\mathrm{SD}}_{G}{+\;\mathrm{SD}}_{R})}{3}}{\mathrm{Max}\;\mathrm{SD}}\;=\;\frac{{(\mathrm{SD}}_{B}{+\;\mathrm{SD}}_{G}{+\;\mathrm{SD}}_{R})}{3\;*\;\mathrm{Max}\;\mathrm{SD}}$$

where SD_B_, SD_G_, and SD_R_ represent the SD of the multispectral bands (blue, green, and red), respectively, and Max SD is the maximum SD value of the three multispectral bands. The OPI values for perfect objects are close to 1, which means that the spectral variation of objects is low and insignificant, while low OPI values show that image objects are heterogenous and their spectral variance is high.

(2) $$\mathrm{OMI}\;=\;\frac{\mathrm{Area}(R\cap S)}{\mathrm{Area}(R)}\;\times \;\frac{\mathrm{Area}(S)}{\mathrm{Area}(R)}$$

where R indicates reference objects and S is a produced object. OMI values less than 1 represent over-segmentation, and values more than 1 indicate under-segmentation. OMI equal to 1 shows that there is a perfect match between both produced object and the reference object.

OFI is used to present object quality based on OPI and OMI values in just a single measure, and it is mathematically stated as follows [57]:

(3) $$\mathrm{OFI}\;=\sqrt{(\frac{{\mathrm{OPI}\;}^{2}{+\;\mathrm{OMI}\;}^{2}}{2}})$$

In OFI, values less than 1 are usually associated with over-segmentation and vice versa.

### 3.4. Artificial Neural Network (ANN)

ANN is an ML mathematical method that is capable of solving complicated and nonlinear problems [58]. An ANN imitates the way a human brain functions [45] by finding patterns and making weighted connections between inputs and outputs in a system [59,60,61], which consists of three layers: the input layer, hidden layer, and output layer. In this study, multi-layer perceptron (MLP) with a back-propagation (BP) learning algorithm, one of the most widely used neural network models, was used [58,62]. This model contains one input layer, one output layer, and one or more hidden layers (depending on the complexity of the problem) to solve complex problems, whereas conventional ANNs usually contain one hidden layer [58].

**Figure 4:**
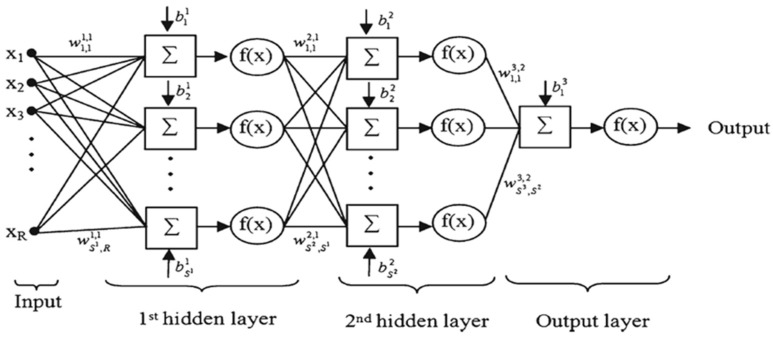
An example of a feed-forward MLP network with two hidden layers. Xs, Ws, bs, and f(x) are inputs, weights, biases, and activation function, respectively.

The MLP was trained by a BP algorithm using a set of training data linked with input and output values (Figure 4). In the MLP network, each input was multiplied by a random weight and then aggregated with a “bias” value, after which it was passed through an activation function to produce the output values. The BA algorithm then trained the network by adjusting the weights in response in order to minimize errors between actual output values and target output values. Following the training stage, the MLP provided a model to predict desired target values based on input values [28,60,62,63,64,65]. The mathematical explanation of the MLP network is as follows:

(4) $$y=\overset{n}{\underset{i=1}{\sum }}\left(W_{i}*X_{i}+b\right)$$

where W denotes the vector of weights, X is the vector of inputs, and b is the bias. The sigmoid activation function, which was used in the present study, is explained as below:

(5) $$f(z)\;=\;\frac{1}{1-e^{-y}}$$

where *f* (z) is the output of activation function, which ranges from 0 to 1 and y is the Equation (1) result. For our case, MLP parameters such as several hidden layers, nodes in each hidden layer, learning rate, momentum factor, and iterations were 2, 10, 0.01, 0.4, and 2000, respectively.

### 3.5. Random Forest (RF)

The RF model is a modern and nonparametric ML classifier that has been used in a wide range of applications like image classification [66], and landslide and gully susceptibility mapping [58,67,68]. This model introduced by Breiman (2001) [69] is based on decision trees, which exploit numerous deeper decision trees for the training process. The result is a diminution of bias and variance [69], thereby making it insensitive to the problem of over-fitting. The simplicity and elucidation capability of the RF model enables the handling of large, complex, and high-dimensional datasets [70] and the performance of classification with a high level of accuracy [67]. The RF model is considered a simplified form of a classification tree approach in the classification and regression trees (CART) model [70]. In the RF model, each decision tree undertakes classification (vote), and the class that gains most votes is selected as the outcome of the model [58]. By using multiple trees, RF is less prone to errors compared to conventional decision trees, and because of random sampling, the correlation between trees is low. The RF model has been reported to provide excellent accuracy in both gully and landslide susceptibility mapping and satellite image classification [58].

In the study reported here, the RF model was selected as one of the ML models to detect gully networks. The number of trees in the RF model was 80, and to specify the split criterion for each node, the square root of several condition factors was used. We the used scikit-learn [71] data mining package in Python environment to run the RF model.

### 3.6. Support Vector Machine (SVM)

SVM is a discriminative ML model known as the maximum-margin model. It was proposed by Vapnik [72] and is one of the ML models applied in this study for detection of gully networks. The model relies on statistical learning theory to overcome intricate problems associated with regression and classification [73,74].

Two classes were separated by mapping the dataset into a higher-dimensional space using non-linear transforms [58], a process that resulted in a hyperplane distinguishing two classes. Also, the hyperplane that presented maximal margins amongst two classes (the optimal hyperplane [75]), and vectors defining this hyperplane were support vectors. Sometimes, finding an optimal hyperplane is difficult or impossible. A soft hyperplane allows training datasets to pass the support vectors, resulting in errors, and training data to cross the margin. The model is, therefore, less accurate and less fitted [26]. Because it was not easy to separate data linearly, a non-linear approach was required in order to handle this problem in regression and classification. Here, the SVM model included both a linear and non-linear kernel to create the hyperplane, because the results depended on the selected kernel function and in order to address classification nonlinearity, we applied a basic radial function (RBF) kernel that has been widely used in remote sensing studies, such as landslide detection [58,65] and gully susceptibility mapping [26]. The RBF mathematical Equation (6) is as follows:

(6) $$K\;\left(x,y\right)=\;\exp(-\gamma \;PX_{i}\;-\;X_{j}\;P^{2})$$

where *γ* is the gamma operator; the way it is estimated influences the results. A gamma operator of 0.9 was selected, and the training data was the same as that used for other models.

### 3.7. Stacking

**Figure 5:**
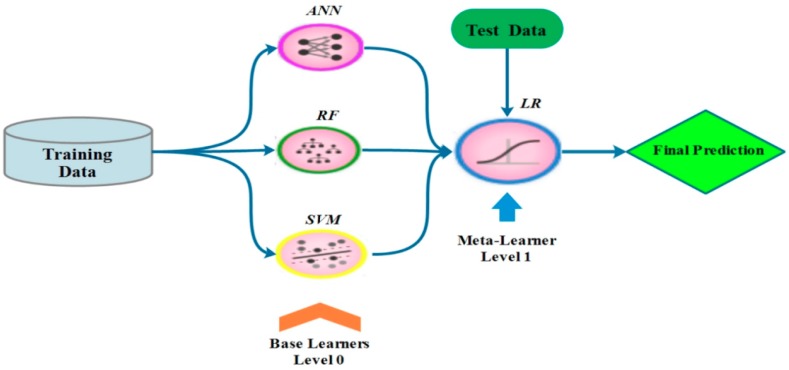
Flowchart of the stacking model.

The stacking model (Figure 5) is one of the current ensembles of ML models introduced by Van Der Leen et al. (2007), [76]. The concept behind this model is to combine weak learners in order to create a powerful model for predicting and classifying a set of data [47,77]. The stacking model includes two levels, namely level 0 and level 1. Level 0 consists of some heterogeneous weak learners such as LR, SVM, and ANN, whereas level 1 only includes one model, called a meta-learner [76] and it can be any learning model. The following steps are required for structuring a stacking model:

- Specify and configure heterogeneous ML models
- Training the learners at level 0 using the training dataset and perform k-fold cross-validation on each of the ML models to collect the cross-validated predicted values of models
- Building an N × M matrix, in which N is cross-validated predicted values from each of the models and M is the learners. This matrix is named “level 1” data
- Fitting a meta-learner model to level-1 data matrix
- Make prediction using trained meta-learner based on the test data

In this study, we used the same training data and parameters used for the other ML models in Level 0. For the meta-learner model, we chose the LR model. In addition, for the applied LR model, for the training and validation processes, we used 10-fold cross-validation. To detect gully features using ML models, the Feature Space Optimization (FSO), which is embedded in eCognition Developer software, is used. It provides the optimal features based on the given training data and object features for image classification and feature extraction. Therefore, before applying ML models, FSO was used to select the optimal features for gully detection. Objects with an overlapping area higher than 50% with gully inventory map (training polygons) were chosen as the training objects to train ML models; the same procedure was implemented to select background training objects.

### 3.8. Accuracy Assessments

**Figure 6:**
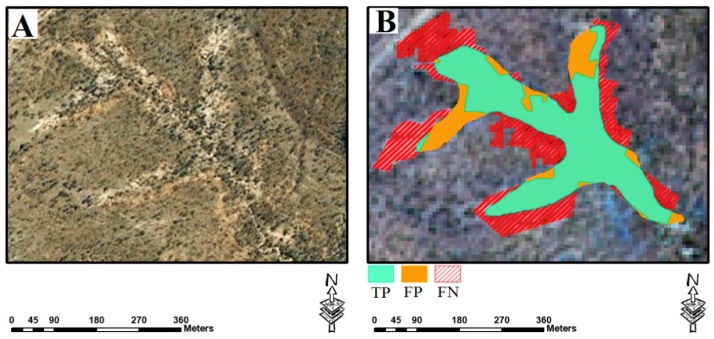
Graphical representation of metrics: TP, FP, and FN. (**A**) is the location of a selected gully feature on Google Earth, and (**B**) is the overlay of TP, FP, and FN of the same gully on Sentinel 2A image.

In any spatial study, it is essential to assess and validate results. Quantitative measures such as Precision, Recall, and F1 measures were employed to validate the resulting maps of the gully networks using the semi-automated object-based approach. An inventory map was used for this aim. Precision assesses how specific models are in identifying gully networks, and Recall shows how many gullies are correctly detected. The F1 measure is a combined measure between Precision and Recall [58]. The measures were calculated based on three metrics, namely true positives (TPs), false positives (FPs), and false negatives (FNs). The TPs indicate the correctly identified gullies, FPs’ metrics represents miss detected gullies, and the gullies that are not detected were labelled as FNs (Figure 6).

(7) $$\mathrm{Precision}=\;\frac{\mathrm{TPs}}{\mathrm{TPs}\;+\;\mathrm{FPs}}$$

(8) $$\mathrm{Precision}=\;\frac{\mathrm{TPs}}{\mathrm{TPs}\;+\;\mathrm{FPs}}$$

(9) $$F1\;\mathrm{measure}=2*\frac{\mathrm{Precision}*\mathrm{Recall}}{\mathrm{Precision}+\mathrm{Recall}}$$

## 4. Results

### 4.1. Image Segmentation

**Figure 7:**
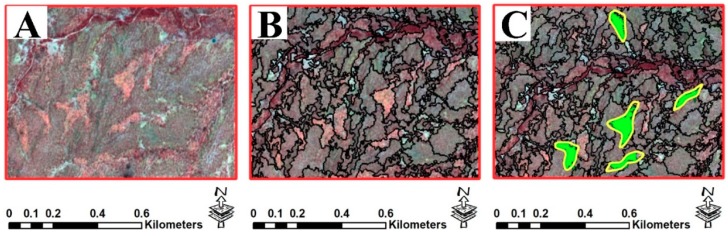
The selection process for image segmentation with scale of 45 and training samples. Part (**A**) is Sentinel 2A false-color composite, part (**B**) is a segmented image, and part (**C**) represents green polygons that are training objects. The yellow polygons are gully inventory maps.

**Figure 8:**
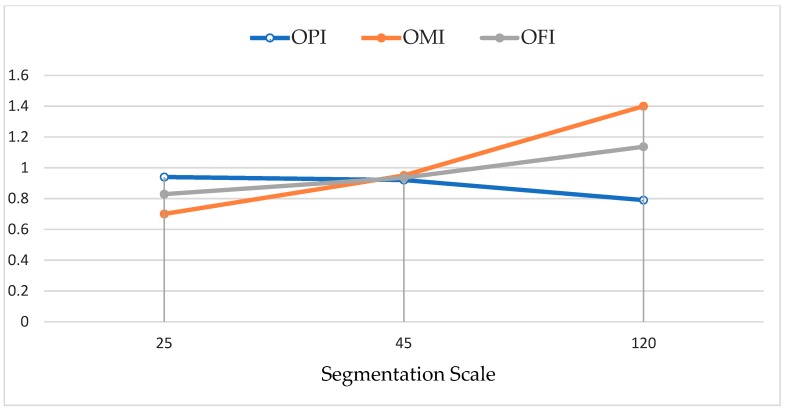
The variation of OPI, OMI, and OFI indices at different segmentation scales. At scale 45, all lines cross each other, showing the best scale value for image segmentation.

The image segmentation process was carried out for the semi-automated object-based approach. We applied the multiresolution segmentation method in eCognition 9.1 based on three scale values calculated by the ESP2 tool to produce image objects from Sentinel 2A images (see Figure 7). During the segmentation process, the same shape index, compactness parameters, and bands weights were used (Table 1). There is no standard and approved method to select the best values for shape, compactness, and band weight; it is based on experimentation, expert knowledge, and background in GEOBIA [78,79]. Since there are no definite patterns and geometrical entities, we decided to allocate equal weights to both shape and compactness. Following other similar studies [79,80,81], weights of two are allocated to NIR and NDVI due to sensitivity of these parameters to water and vegetation cover [80]. The segmentation results, using the scales of 25, 45, and 120, were evaluated using OPI and OMI indices based on a gully inventory map (Table 1 and Figure 8).

In pixel-based models, pixel values in corresponding layers are evaluated, but because they have no dimensions, other geometric and textural characteristics cannot be used. In object-based methods, an image object is a two-dimensional feature, and thus a wide range of geometric, textural, and contextual characteristics can be extracted from each object.

### 4.2. Gully Network Detection Using ML and Stacking Models

**Figure 9:**
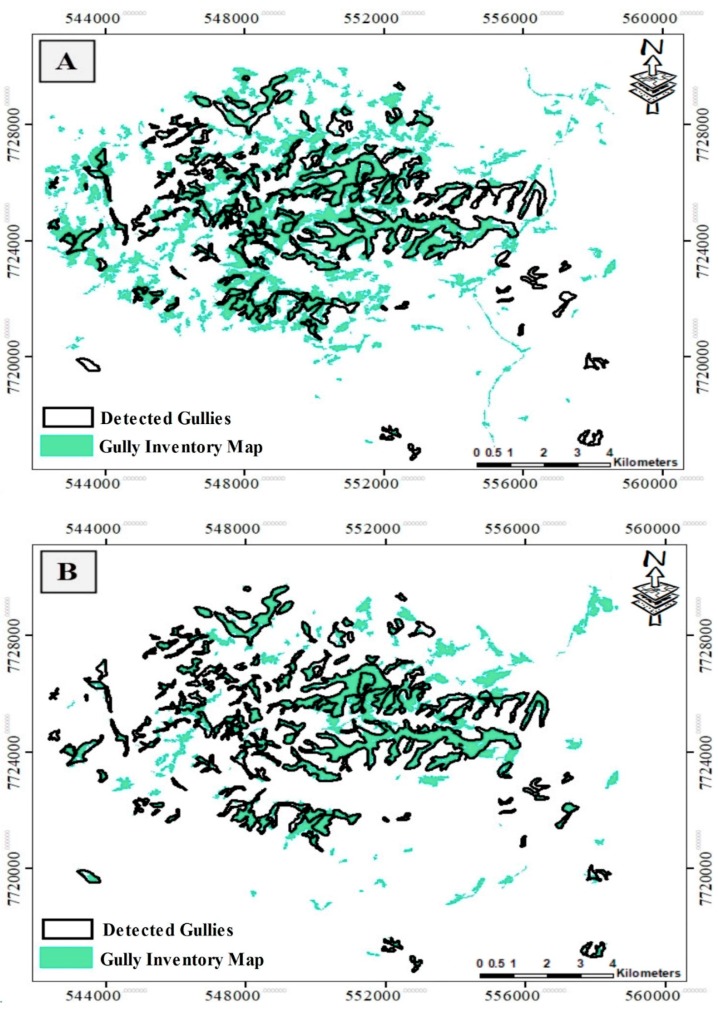

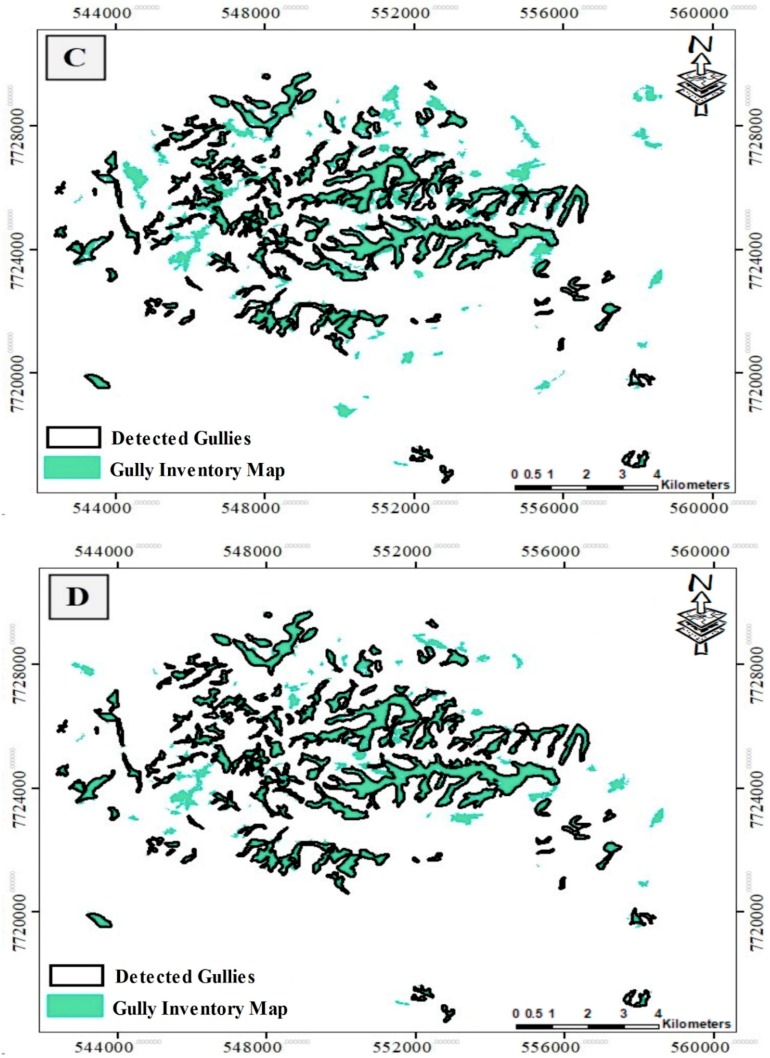
The results of gully networks detection based on segmentation scale 45: (**A**) SVM, (**B**) ANN, (**C**) RF, and (**D**) stacking model. The black color indicates detected gullies, and cyan polygons represent the gully inventory map.

Using FSO mean values for conditioning factors for each object, characteristics such as shape index, length-to-width ratio, density, compactness, NDVI standard deviation, slope standard deviation, drainage density standard deviation, brightness, grey-level co-occurrence matrix (GLCM) correlation, GLCM contrast, and GLCM dissimilarity were selected with separation distance of 3.2 as the optimal input features for gully networks detection. The FSO result indicates that using all input features together in ML models does not guarantee the best outcome; thus, it is required that the most suitable features are selected. To apply ML models on image objects based on each scale, training and test dataset were stored in a comma-separated values (CSV) format and imported into the Python environment to train and run models on test data. The parameters of single ML models and base learners in the stacking models were the same. K-Fold cross-validation with “k” of 10 was used for all models. The k-fold cross-validation was used for dealing with the randomness effects of the training and testing inventory selection on the performance of the ML models [34]. Thus, the inventory map was randomly divided by 10, and the folds used for training and testing were changed ten times; the average value of all ten folds was represented in this study. The values varied from 0 to 1; values close to 1 showed the highest probability of being a gully feature, whereas values close to 0 indicated non-gully features. All objects that had values greater than 0.5 were classified as gully networks. In Figure 9, the maps are based on the optimal segmentation scale and the best result from the 10-fold cross-validation on the ML models.

The stacking model provides the best result compared to other models, whereas SVM performs poorly in gully networks detection. Although all single models were able to detect gullies to some extent, the degree of missed gullies was celebrated in the stacking model—it was the lowest in comparison with other models. More details of model accuracy are discussed in the next section.

In order to evaluate the reliability of each model, a gully detection map was used to evaluate the models’ performance. According to Table 2, the validation results showed that the SVM model had the lowest accuracy and weakest performance in gully networks detection (precision 0.67 and recall 0.71), whereas the stacking model (precision 0.84 and recall 0.95) had the highest accuracy and performance in mapping gully networks. The ANN model had a relatively better performance compared to the SVM model but weak performance in comparison with the RF and Stacking models. Furthermore, the RF accuracy assessment result (precision 0.81, recall 0.9) was considerably better in detecting gullies in comparison with the SVM and the ANN models. Although the values of precision and recall for the RF model were lower than those of the stacking model, it still provided proper detection. In all models, the area of missed detected gullies (FPs) and undetected gullies (FNs) was high, except for the stacking model. For example, in the SVM model, FPs were approximately 599 ha, which is nearly equal to one-half of the correctly detected gullies and almost equal to the gullies that were not detected, an outcome for ANN that was almost the same. The area of FPs in both RF and stacking models was around one-fifth of the accurately mapped gullies, but the area of undetected gullies was 85 ha in the stacking model, substantially lower compared to RF and the other models. Although the stacking model had high FPs, the FNs’ value was the least compared to the other models, and it almost detected all gullies. We compared the results of this study with similar studies in gully detection carried out by others. In a study by d’Oleire-Oltmanns et al. [37], gullied areas were mapped by applying a rule-based object-based image classification method on QuickBird 2 images with panchromatic and multispectral resolution of 0.6 m and 2.4 m, respectively. Although in this case, very high-resolution images were used, the gully mapping result with 62% overall accuracy and 38% omission error was not satisfying because the classification rules were based on expert knowledge, not trained ML model. However, in our study, the Sentinel 2A data with lower spatial resolution resulted in better results due to selection of the best segmentation scale and using ML models. In another study by Shruthi et al. [68], ASTER satellite images were used along with object-based random forest model for gully mapping. ASTER multispectral and stereo-pair images with 15 m spatial resolution were employed to produce spectral and topographic variables for segmentation and object classification. The optimal scale was obtained by the ESP tool, but the accuracy of image objects was not assessed. Gully detection results based on the RF model provided similar accuracy (81%) to our study. Compared to the two above-mentioned studies, our results are more reliable due to the application of measures such as OPI, OMI, and OFI, as well as the use of ML methods (like the stacking model).

## 5. Discussion

This study aimed to integrate GEOBIA with ML models in order to deal with scale problems in object definition for detecting gully networks. We evaluated the performance of different ML models at a site in the north part of the Bowen catchment, Australia, with a high density of gully networks. The mapping of gully networks and their development over time is considered a crucial requirement for estimating the soil erosion and sediment outlet to the world heritage Great Barrier Reef. The resulting detailed gully network maps are also applicable to predict vulnerable regions with regard to gully formation, land degradation, and adverse impacts on the environment. Over the past two decades, the GEOBIA method has become the conventional approach in image classification and feature detection of landslides [56,82,83,84,85,86,87] and gullies [35,37,39,54,68]. The current study used similar object-based gully detection approaches, but with a different approach—reconsideration in selection of the best segmentation scale parameters and ML models. The complexity in image segmentation usually produces wrong results. Thus, in some studies, different scales are used in segmentation, to produce objects that are applied to further classification tasks. Karami et al. 2015 [35] applied both pixel and object-based classification approaches for gully networks mapping. In order to apply OBIA and improve its accuracy and effectiveness for multi-scale segmentation, three different scales of 20, 30, and 60 were used. Although the authors claimed that an RF model with scale of 60 provided the best accuracy, the impact and accuracy of segmentation scales are unclear. In this study, the ESP2 tool was used to find three optimal scales (fine to coarse) for image segmentation. Then using OPI, OMI, and OFI, the accuracy of each scale was assessed on the basis of a gully inventory map. The accuracy assessment results showed that segmentation with scale of 45 provided objects with the highest accuracy. The accuracy of results may relate to the polygon sizes of the gully networks, regardless of the selected scale. The optimal scale in an area with specific gully polygons size may result in different optimum scales than neighboring area with different polygon sizes. However, the same methodology is completely transferrable and can be applied elsewhere.

Conventional pixel-based ML models are limited to pixel values, whereas in this study we were able to capitalize on several object-based method advantages, including geometric and textural characteristics (e.g., shape index, standard deviation, GLCM, etc.). However, we cannot directly use all statistical and geometrical features (33 items) together for object classification and feature detection. It is essential to choose features that provide the best results, and in this case, this task was done by FSO, identifying 23 features (Section 4.2). Although we used topographic factors such as TWI and slope length as input features, after applying FSO, these factors were excluded from the final gully network detection model.

Here, we implemented SVM, ANN, RF, and ensemble (stacking) models in a Python environment. Conventional GEOBIA ML models are usually used individually and their results are then compared. In this case, ML models are used as combined and ensemble (stacking) form. Among the applied models, SVM showed the weakest performance and lowest accuracy in gully mapping. The SVM model had the highest rate of misdetection (34%) relative to the gully inventory area, whereas the stacking model provided less than 18% in misdetection. However, in detecting actual gully networks, stacking detected almost 95% gullies and SVM was the lowest (0.71% of gully networks). Topographic derivatives used as conditioning factors influenced ML results, in particular, their spatial resolution. The conditioning factors were extracted from coarse resolution 12-m ALOS-2 DEM. Using high resolution or very high resolution data (i.e., sub-meter topographic data) can potentially improve image segmentation, as well as the ML models’ accuracy. Finally, this study showed that the integration of GEOBIA and ML models, in particular the stacking model, on high-resolution data such as satellite images and topographic data could show robust performance in the process of detecting and mapping features such as gullies.

## 6. Conclusions and Future Work

Gully erosion is considered a significant cause of soil erosion and land degradation globally, and needs to be both detected and accurately predicted. The Burdekin Basin in Australia is affected by gully erosion and is a major source of sediment to the GBR, degrading the health of the reef [20]. In this study, we integrated four different ML models with GEOBIA to map gully networks in the Bowen catchment, a highly gullied sub-catchment of the Burdekin Basin. With regard to the assessment of accuracy, the integration of optimal segmentation from GEOBIA with the model provided the best performance, when compared to other single models. Although the integration of GEOBIA and ML models were useful in gully mapping, aspects such as selection of conditioning factors and image segmentation quality should be carried out with care. In future work, we will apply extra gully erosion conditioning factors, such as a connectivity index and a rainfall erosivity map, which were not available for this study. We will evaluate the related probability concepts by considering spatial homogeneity in order to deal with the scaling problem for object definition in GEOBIA. Related probability concepts such as central limit theorem and Dempster–Shafer theory will be used to detect an appropriate scale for image segmentation.

## Figures and Tables

**Table 1:** Segmentation parameters (optimal scale parameters are in bold) and the results of object evaluation indexes.

Scale	Shape	Compactness	Layer Weights					OPI	OMI	OFI
			Blue	Green	Red	NIR	NDVI			
25	0.5	0.5	**1**	**1**	**1**	**2**	**2**	0.94	0.70	0.83
**45**	**0.5**	**0.5**						**0.92**	**0.95**	**0.94**
120	0.5	0.5						0.79	1.40	1.14

**Table 2:** The quantitative accuracy assessment of applied machine learning (ML) models in gully networks detection.

	TP (ha)	FP (ha)	FN (ha)	Precision	Recall	F1 Measure
SVM	1237	599	512	0.67	0.71	0.69
ANN	1375	486	374	0.74	0.79	0.76
RF	1566	374	183	0.81	0.90	0.85
Stacking	1664	325	85	0.84	0.95	0.89
